# A Psychophysical Investigation of Differences between Synchrony and Temporal Order Judgments

**DOI:** 10.1371/journal.pone.0054798

**Published:** 2013-01-21

**Authors:** Scott A. Love, Karin Petrini, Adam Cheng, Frank E. Pollick

**Affiliations:** 1 School of Psychology, University of Glasgow, Glasgow, United Kingdom; 2 Department of Psychological and Brain Sciences, Indiana University Bloomington, Bloomington, Indiana, United States of America; 3 Institute of Ophthalmology, University College London, London, United Kingdom; Nothwestern University, United States of America

## Abstract

**Background:**

Synchrony judgments involve deciding whether cues to an event are in synch or out of synch, while temporal order judgments involve deciding which of the cues came first. When the cues come from different sensory modalities these judgments can be used to investigate multisensory integration in the temporal domain. However, evidence indicates that that these two tasks should not be used interchangeably as it is unlikely that they measure the same perceptual mechanism. The current experiment further explores this issue across a variety of different audiovisual stimulus types.

**Methodology/Principal Findings:**

Participants were presented with 5 audiovisual stimulus types, each at 11 parametrically manipulated levels of cue asynchrony. During separate blocks, participants had to make synchrony judgments or temporal order judgments. For some stimulus types many participants were unable to successfully make temporal order judgments, but they were able to make synchrony judgments. The mean points of subjective simultaneity for synchrony judgments were all video-leading, while those for temporal order judgments were all audio-leading. In the within participants analyses no correlation was found across the two tasks for either the point of subjective simultaneity or the temporal integration window.

**Conclusions:**

Stimulus type influenced how the two tasks differed; nevertheless, consistent differences were found between the two tasks regardless of stimulus type. Therefore, in line with previous work, we conclude that synchrony and temporal order judgments are supported by different perceptual mechanisms and should not be interpreted as being representative of the same perceptual process.

## Introduction

One of the main techniques of multisensory integration research involves manipulating the temporal relationship of crossmodal cues to an event, and examining the consequences [1]. The responses to two main behavioural tasks have been used as dependent measures in such research with human participants: synchrony judgment (SJ) and temporal order judgment (TOJ). These are not the only tasks used in synchrony perception research (e.g., [2]–[4]) but they are the focus of the current work. SJs involve participants deciding whether two sensory cues (e.g., audio and visual) to a bimodal event (e.g., audiovisual speech) are in or out of synch, whereas in TOJs participants decide which cue came first or second. In the late 1950s, however, Hirsch [5] argued that, in relation to unimodal visual TOJs, the two tasks do not measure the same perceptual process. Early evidence that the tasks differ for audiovisual stimulation can be found in the work of Allan [6], who argued that while perception of successiveness/asynchrony was sufficient for participants to make SJs, this was not the case for TOJs. Despite this proposal [6], parameters of interest derived from these tasks were still regularly given the same names (PSS and TIW – defined below), which could lead to their being interpreted as representing the same perceptual process. It is this concern that prompted recent studies to again explore whether or not the two tasks significantly differ (e.g., [2], [3], [7]–[10]).

One of the most obvious indicators that the underlying mechanisms of SJs and TOJs are not equivalent is a large difference in the point of subjective simultaneity (PSS) derived from these two tasks [7], [8], [10]. The PSS is defined as the amount of asynchrony (usually in milliseconds) between the two cues that most often results in them being perceived as synchronous. A recent project designed to investigate differences between SJs and TOJs focused on inconsistent PSS values derived from the two tasks [2]. The authors' extensive review of the literature highlighted that for both tasks, both audio- and video-leading PSSs have been observed in individual participants, yet, and importantly, *mean* audio-leading PSSs are “almost exclusively” reported for TOJs, whereas SJ *mean* PSSs are generally video-leading [2]. Audio-leading PSSs are regarded as highly unnatural because, in natural situations, auditory cues generally lag visual cues (e.g., [2]). For example, in face-to-face conversation the auditory cue to speech can actually begin anywhere between 100 and 300 ms [11]–[14] after the initiation of the facial movements (the visual cue to speech). Interestingly, in their investigation of differences between SJs and TOJs using the same audiovisual stimuli and participants, Van Eijk et al. [2] did not find an overall audio-leading TOJ PSS. Neither did they find a significant difference between their SJ and TOJ PSSs for a simple stimulus (flash-click). Their strong conclusion, that it is wrong to use the same terms for the parameters (e.g., PSS) derived from the two tasks, was based on a difference in PSS only for the more complex stimulus tested (bouncing ball) plus an overall lack of correlation between SJ and TOJ PSSs (see, [9] for a similar lack of correlation using speech stimuli).

It is interesting that reported differences and commonalities between the task parameters were dependent on stimulus type (flash-click or bouncing ball). The hypothesis that people may have different sensitivities to asynchrony for different stimulus types has been corroborated by many studies (e.g., [15], [16]), while it is also becoming apparent that these differences may not be the same across SJs and TOJs [2], [7]. Van Eijk et al. [2] pointed out that even their more complex stimulus lacked ecological validity, hence one of the main aims of the current experiment was to test whether any differences between the two tasks are consistent across a variety of stimulus types.

Using a variety of stimulus types also allowed us to explore a potential confound in previous work. Studies concluding that SJs and TOJs are supported by different perceptual mechanisms may be confounded by the fact that they used stimuli in which duration (onset of the first cue until offset of the second cue) increased linearly with increasing COA [2], [3], [9], [10]. For such stimuli it is possible that participants could correctly make SJs based solely on the duration of the stimulus, i.e., longer durations are asynchronous, while duration provides no information to aid in making TOJs. Two of the five stimulus types used in the current experiment (beep-flash-drumming and point-light-drumming) overcome this confound by having a constant duration that does not increase with increasing COA (see Methods). Hence, those stimuli allowed us to explore whether differences between the tasks occurred solely due to this confound.

In synchrony perception research, a measure of how sensitive participants are to cue onset asynchrony (COA) is used to define the temporal integration window (TIW). The TIW can be derived from the standard deviation, or just-noticeable-difference, of data fits centred on the PSS. It is considered to represent the temporal window, or range of tolerance, of audiovisual asynchrony, within which the perceptual system integrates the cues and prevents reliable detection of asynchrony or cue order. In other words, during an SJ task, stimuli that are physically asynchronous will generally be perceived as being synchronous if they are within the TIW. The idea that during a TOJ task stimuli within the TIW are also generally perceived as synchronous can also be held under the assumption that responses near chance level, i.e., around the PSS, actually correspond to the perception of synchrony. Even without this assumption the width of the TIW represents a measure of sensitivity to the same independent variable, i.e., the COA, in both tasks. Therefore, comparing the TIW, especially of the same group of participants under the same stimulus and experimental conditions, across the tasks is a good way to measure differences in sensitivity to COA between them. However, statistical tests comparing differences between SJ and TOJ TIWs have rarely been reported, even in those studies directly comparing the tasks [2], [8]–[10] – although see [7]. In one exception, Soto-Faraco and Alsius conducted two separate experiments, using different participants but similar audiovisual speech stimuli and design [17], [18] and reported a significantly larger TIW for TOJs than SJs. However, it is not clear whether this result would have held if the same participants had been used for both tasks.

The synchrony perception literature contains many examples of participant data being treated as ‘noisy’ and removed from further analysis due to unacceptable data fits; this is the case for both TOJ (e.g., [19]–[28]) and SJ (e.g., [17], [29]–[31]). For TOJs, data exclusion rates have been as large as ∼35% on more than one occasion [22], [24], [27], while the highest we are aware of for SJs is 26% [29]. While data exclusion is justifiable on many occasions, the regular occurrence of, and high rates of, data exclusion in synchrony perception research have led us to believe that it may reflect something more than noisy data. Furthermore, large data exclusion rates ultimately produce biased mean estimates of performance by ignoring the data of those participants who are unable to achieve the task with the COA levels presented.

Data exclusion rates should be particularly informative when comparing the performance of the same participants on TOJs and SJs under the same stimulus and experimental conditions – random responses in one task compared to more accurate responses in the other would highlight different task demands. Under such circumstances, Petrini and colleagues [8] could only acceptably fit a cumulative Gaussian function to the TOJ data of approximately 50% of their participants, compared with fitting 100% of SJ data for the same participants with a Gaussian probability density function. This result can be interpreted as reflecting the fact that some participants were considerably less accurate in making TOJs than SJs to the same stimuli under the same experimental settings. However, an alternative interpretation would be that the cumulative Gaussian function was not the most appropriate function to fit the TOJ data. If we accept the first interpretation, it would be prudent to treat data exclusion rates as an outcome measure rather than simply as noisy data. Moreover, data exclusion can be related to the TIW by highlighting that as the TIW increases, fitted functions become flatter, up to a point when the TIW is so wide that the data cannot be acceptably fit. In this way, excluded data can be regarded as representing an overly large TIW for the COA levels presented. The points raised above led us to investigate whether participants are less sensitive to COA during a TOJ task than during an SJ task due to different task demands. We explored this point in three ways: by comparing the amount of excluded data across tasks, by comparing TIWs across tasks and by asking participants which task they found “most difficult”.

The current experiment involved the same group of participants making both audiovisual SJs and TOJs, in separate blocks, to five different stimulus types presented in separate experimental runs. The overall aim of the experiment was to provide more psychophysical evidence for the proposal that SJs and TOJs involve different perceptual mechanisms and to further explore whether this would be consistent across a variety of the stimulus types generally used in synchrony perception research. Based on our review of the literature, our experiment was designed to explore several hypotheses: 1) participants will be less sensitive to COA during a TOJ task than during an SJ task; 2) TOJ PSSs will be unnaturally audio-leading, while SJ PSSs will be video-leading and; 3) neither PSS nor TIW values will correlate across the two tasks.

## Materials and Methods

### Participants

28 participants (14 female, age range  = 19 to 32, mean  = 22.9 years) took part in the present study. All were native English speakers (except one, who described their first language as English), had normal or corrected to normal vision and reported no hearing difficulties. Participants were screened for musical abilities prior to taking part: only those individuals with no drumming experience, less than two years professional training on any instrument and a minimal amount of self-tuition were allowed to take part. Participants gave informed written consent and were paid for participation. The University of Glasgow ethics committee approved the experiment and it was conducted in accordance with the ethical standards laid down in the 1964 Declaration of Helsinki.

### Stimuli

Five stimulus types were used: 1) beep-flash (BF), 2) beep-flash-constant-visual (BFV), 3) beep-flash-drumming (BFD), 4) point-light-drumming (PLD) and 5) face-voice (FV).

**Figure 1:**
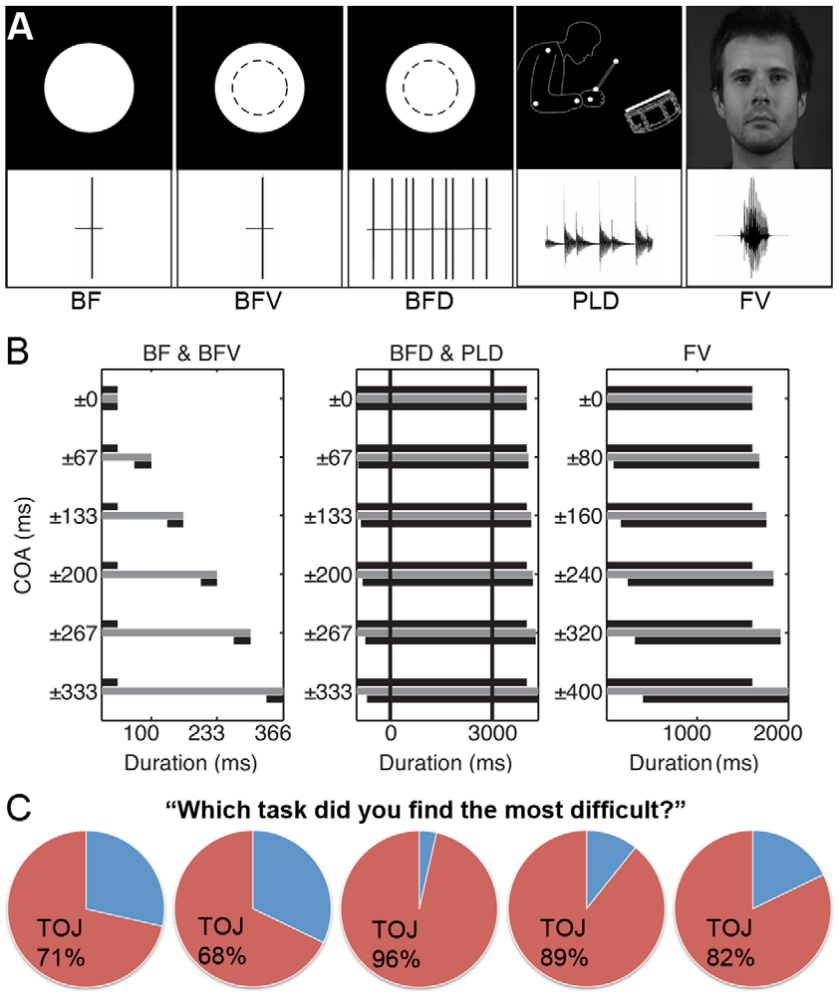
Stimulus illustrations, timing characteristics and subjective impressions of task difficulty. (**A**) Top and bottom rows illustrate the visual information and auditory waveform for each stimulus, respectively. (BF) beep-flash stimulus, consisted of a single flash of a white dot on a black background and a single beep. (BFV) beep-flash-constant visual, was the same as (BF) except there was a smaller white dot, illustrated by the dashed circle, constantly on screen. (BFD) beep-flash-drumming, was the same as (BF) except there were 9 beep-flashes in each stimulus. (PLD) point-light-drumming, shown is a single movie frame and the waveform drumbeat. (FV) A single frame from the face-voice movie and the waveform representation of the word ‘tomorrow’. Note, the images are not to scale, the area of the point-light-drummer and the white flash dots (BF, BFV & BFD) approximately subtended the area of the mouth region in FV. (**B**) Illustration of timing characteristics (onset, offset and duration) for BF and BFV (left), BFD and PLD (middle) and FV (right). COA levels (ms), both audio- and video-leading, are displayed on the y-axis and the x-axis represents duration (ms) – note that x-axis scales are different across the 3 figures. For each COA level gray bars represent overall stimulus durations (see *Stimuli* section for details), while black bars represent relative timing characteristics between audio and video sequences. Top and bottom black bars can represent either the audio or video sequence. For example, the top black bar of the 67 COA condition for BF would represent the audio sequence for an audio-leading condition, while it would represent the video sequence for a video-leading condition. Vertical black bars in the middle figure (BFD &PLD) highlight that the duration of all COA stimuli was 3s and that the stimuli were cut from a larger audiovisual movie after separating the audio and video cues in time by each COA. (**C**) Separately for each stimulus type, pie charts represent the percentage of participants who found either SJ or TOJ the most difficult. Order of pie charts matches the order of the stimuli presented above in (A).

1. In BF stimuli the beep was a pure tone at 2000 Hz and 84 dB mean intensity, while the flash was a white dot (luminance: 85 cd/m^2^) presented on a black background (luminance: 0.12 cd/m^2^, see Figure 1A for an illustration and Movie S1). The area of the white dot (visual angle of the diameter was 4.4 degrees) approximated the area subtended by the drummer and the speaker's mouth region in the PLD (4) and FV (5) displays, respectively. To produce the audiovisual movies (60 Hz, see Movie S1), the pure tone and white dot were imported in Adobe Premiere 1.5 and their duration was resized to 33 ms to create the synchronous (0 ms COA level) condition. Separating the audio and video timelines in 4 frame increments created 11 COA levels: 5 audio-leading (−333, −267, −200, −133, −67 ms), 5 video-leading (+333, +267, +200, +133, +67 ms) and 1 synchronous. A black screen with no sound was used to fill the gap between the beep and flash in the ten asynchronous conditions. Stimulus duration of the synchronous condition was 33 ms, while the duration of asynchronous conditions increased with increasing COA: 366, 300, 233, 166, 100 ms respectively for the ±333, ±267, ±200, ±133, ±67 ms COA conditions. The left panel of Figure 1B illustrates the relative timing characteristics (onset, offset and duration) of the audio and video sequences as well as overall stimulus duration.
2. BFV stimuli had the same properties as the BF except there was a constant white dot (visual angle of diameter was 2.5 degrees) on screen, which increased in size, to 4.4 degrees, to produce the flash (Figure 1A, left panel of Figure 1B and Movie S2).
3. BFD stimuli had the same visual and auditory properties as BFV (Figure 1A and Movie S3); however, the auditory and visual sequences contained 9 beep-flashes, the timing properties of which mimicked the swing groove drumbeat of the PLD stimuli described below, i.e., at times when a drumming impact would occur in PLD a beep-flash occurred instead. The COA levels (±333, ±267, ±200, ±133, ±67, 0 ms) and the relative timing characteristics (onset, offset and duration) of the audio and video sequences were the same as those in PLD (middle panel of Figure 1B).
4. Detailed description of the PLD stimuli has been published elsewhere [8], [32], [33]. Here we describe aspects of the stimuli important to the current experiment. Stimuli were dynamic audiovisual movies (60 Hz) containing the point-light representation (Figure 1A and Movie S4) of a drummer playing a swing groove at 120 beats per minute and accent on the second beat. The full image covered a visual angle of 4.8 degrees wide and 2.8 degrees in height. Both synchronous and asynchronous PLD stimuli were cut from a 15 s original recording to contain 9 audio and visual impacts [32]. Cutting the stimuli from a longer drumming sequence after separating the audio and visual cues in time by each COA level (±333, 267, 200, 133, 67, 0 ms) enabled there to be audio and video sequence at the beginning and end of all 11 COA stimuli (Figure 1B middle panel). Hence, this technique of creating asynchronous stimuli contrasted with the method used for BF, BFV and FV, in which a separation of the audio and visual cues produced gaps at the beginning and end of the stimuli. Moreover, while stimulus duration increased with increasing COA level for BF, BFV and FV (left and right panels of Figure 1B) it was a constant 3 seconds for every COA level for PLD and BFD (middle panel of Figure 1B). Both techniques for creating asynchronous conditions have been extensively used in other published work (e.g., [1], [8], [16], [32]).
5. FV stimuli were dynamic audiovisual movies (25 Hz) of a native English speaker saying “tomorrow”. The visual speech cue contained the full face and covered an approximate visual angle of 12.7 by 18.9 degrees (Figure 1A and Movie S5); the mouth region subtended approximately 3.2 by 2.5 degrees. To produce asynchronous conditions the audio and visual streams were separated along the movie timeline relative to each other using a method similar to previous research [16]. This separation produced gaps at the beginning and end of the movie timeline, which were appropriately filled with the first and last frame of either the auditory or visual stream to produce a non-speaking still face image. For speech stimuli, previous work (e.g., [30], [31], [34]) used a wider range of COA levels than that of our stimuli described above; hence, we used a wider range for our face-voice stimuli. Ten asynchronous versions were created with the audio stream shifted to begin either before the video stream (−400, −320, −240, −160, −80 ms) or after (+400, +320, +240, +160, +80 ms), in 80 ms (2 frames) increments. Similar to BF and BFV, stimulus duration can be calculated by adding the COA level to the duration of the synchronous condition (1.6 s); hence, duration ranged between 1.6 seconds for the 0 COA condition and 2 seconds for the ±400 ms COA conditions (right panel of Figure 1B).

These stimuli were chosen as they represent a variety of the types of stimuli generally used in synchrony perception research. Interestingly, there are differences in complexity across the stimuli: As the BF and BFV conditions contain only a single circle of light and a beep they can be regarded as simpler than the BFD condition, which contains the same visual and auditory characteristics but presented in a rhythmical sequence; PLD is more complex than BFD because it contains the point-light representation of the natural motion characteristics of a human arm drumming, and FV is the most complex as it contains the audiovisual information of a natural video recording of the talking human face. Differences in PSS and TIW have previously been shown across these general types of stimuli (e.g., [15], [16], [32], [35]); hence, it was of interest to test if any differences found between TOJs and SJs were consistent across these stimulus types [2]. Moreover, due to the different methods of creating asynchrony described above the duration of BFD and PLD was a constant 3 seconds, while the duration of BF, BFV and FV all changed with increasing COA. Therefore, using these multiple stimulus types also enabled us to investigate a possible confound in previous work [2], [3], [9], [10]: differences between the tasks highlighted using only stimuli in which duration increased with increasing COA could simply reflect that SJs but not TOJs can be correctly made based on stimulus duration alone.

### Apparatus and Procedure

Stimuli were presented via an Apple Macintosh MacPro 3.1 desktop computer running OS ×10.5 and an NVIDIA GeForce 8800GT video card. The visual cues were displayed on a 21-inch ViewSonic Graphics Series G220f CRT monitor running at 1024×768 screen resolution and 60Hz refresh rate. Auditory cues were presented through high quality headphones (Bayerdynamic DT770). Presentation was achieved using MATLAB 2007b (MATHWORKS Inc., Natick, MA) and the Psychophysics Toolbox (PTB3) extensions [36], [37].

The experiment was split into 5 sub-experiments, one for each stimulus type. The order of these was pseudo randomised for each participant, with an attempt to have a similar number starting on each stimulus type: 5 started on BF and BFV, while 6 started on the other three stimulus types. The 5 experiments were split across 2 sessions, each approximately 1-hour, which were completed on separate days to reduce the effects of fatigue (mean separation  = 3.1 days). Each experiment presented only one stimulus type and consisted of 24 blocks: half of the blocks were SJ blocks and the other half were TOJ, presented in a randomised order. After each sub-experiment participants completed a debrief questionnaire, which included the question, “Which task did you find the most difficult?”

The experiments took place in a quiet darkened room in which participants sat approximately 90 cm from the stimulus display monitor. At the start of each experiment, participants were given written instructions, completed 6 practise trials (3 SJ and 3 TOJ) and asked any questions of clarification before the experimenter left the room. Participants then pressed any key to begin the experiment and the instructions as to whether the first block was an SJ or a TOJ block appeared on screen for 4 seconds. The relevant task instructions were presented for 4 seconds at the start of every block. Within a block there were 11 trials: one presentation of each COA level of the current stimulus type. Participants had to base their SJ and TOJ judgments on the entire stimulus duration and could only make a response once the stimulus had finished and the possible responses were displayed on screen: after each trial the current task question and possible answers were displayed on screen until the participant responded, which triggered the start of the next trial. During SJ blocks participants were instructed to press ‘1’ or ‘3’ on the number pad depending on whether they thought the audio and visual cues were synchronous or asynchronous, respectively. During TOJ blocks they pressed ‘1’ if they thought the video came first and ‘3’ if they perceived the audio to come first. No feedback was given. In total there were 12 trials per COA level for each task/stimulus combination. A similar number of trials have been used in other related work [16], [38], and Petrini et al [8] reported no significant difference between the results of 10 or 20 trials.

### Analysis procedure

For SJ/stimulus combinations the proportion of synchronous responses at each COA level were fit with a Gaussian probability density function, while for TOJ combinations the proportion of video first responses were fit with a Gaussian cumulative distribution function (for similar methods see, [8], [10]). This fitting procedure was conducted separately for each participant and task/stimulus combination. Two parameters of interest were derived from these fits: the point of subjective simultaneity (PSS) and the temporal integration window (TIW). The PSS represents the level of COA that participants perceived as most synchronous; it was taken as the maximum of the best-fitting SJ curve and the 50% point from the TOJ curve. The TIW represents the range of COA, centred on the PSS, within which participants could not reliably perceive asynchrony or cue order, and this was defined by the standard deviation of each best-fitting Gaussian [8], [35].

To examine whether on average participants were better at detecting audio-leading than video-leading COA we carried out a bootstrap analysis [39] on the percentage correct data at each asynchrony level. Separately for each task/stimulus combination we resampled, with replacement, those participants not excluded from the combination. To produce a distribution of mean percentage correct at each asynchrony level, we created 10,000 bootstrapped data sets for each combination. This distribution was used to calculate 95% confidence intervals for the mean percentage correct at each asynchrony level. No overlap in these intervals represents a significant difference between the audio and video leading conditions [39].

## Results

The current experiment involved participants (N = 28) making either SJs or TOJs, in separate blocks, to one of five different stimulus types (Figure 1A) presented in separate experimental runs. The data from each participant was fitted with a psychometric function separately for each task/stimulus combination (e.g., TOJ/BF, SJ/BF). For SJs the proportion of synchronous responses at each COA level were fit with a Gaussian probability density function, while for TOJs the proportion of video first responses were fit with a Gaussian cumulative distribution function.

### Individual data fitting and Sensitivity to COA for SJs and TOJs

Our first hypothesis stated that participants would be less sensitive to COA during a TOJ task than during an SJ task and we explored this hypothesis in three ways. First, to assess participants' subjective impression of a difference in sensitivity to COA between the tasks, after completing each task/stimulus combination they expressed which task they found the most difficult. For every stimulus type the majority of participants found the TOJ task to be more difficult than the SJ task (Figure 1C).

**Figure 2:**
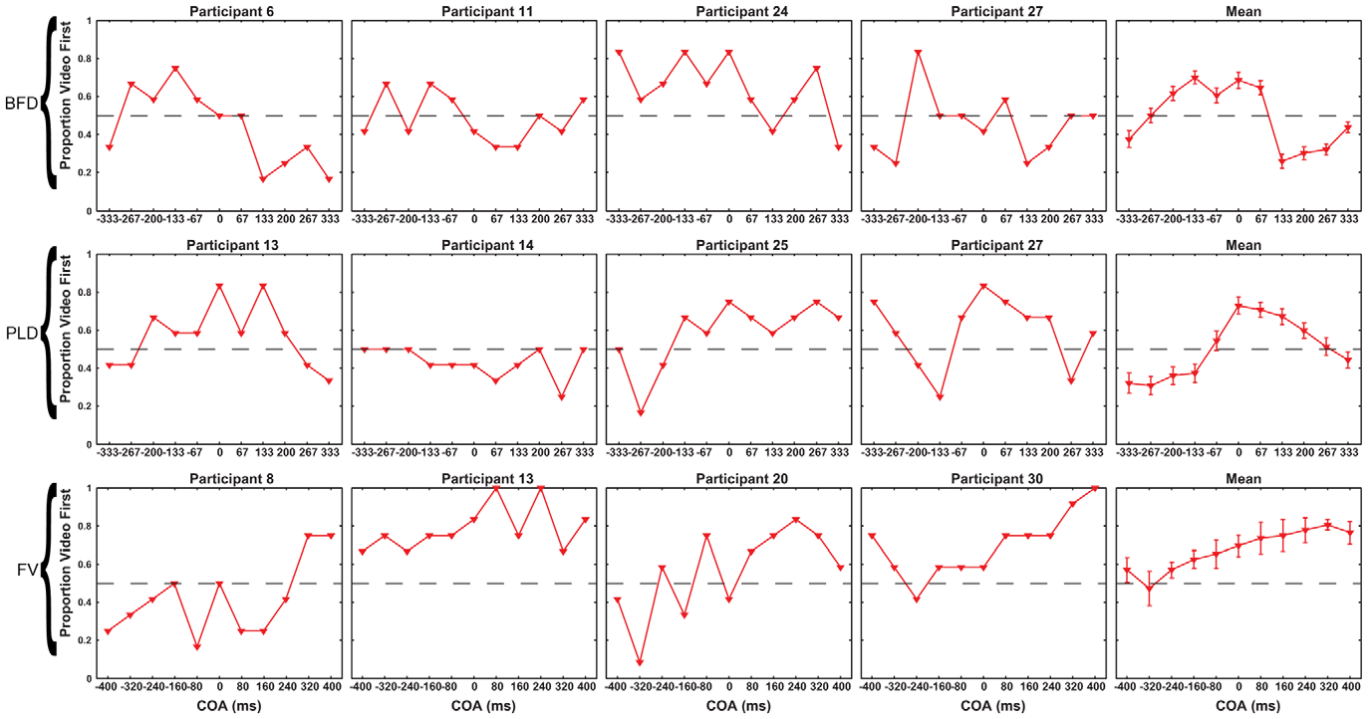
Examples of excluded TOJ data for BFD, PLD and FV stimuli. Red triangles represent the proportion of video first responses at each COA level. Each row displays 3 randomly selected excluded data sets plus the mean of the excluded data for a particular stimulus type (top for BFD, middle for PLD and bottom for FV). The dashed horizontal line represents chance performance. Errorbars are ± one standard error of the mean. BF and BFV conditions are not included as no TOJ/BF data was excluded and only a single subject was excluded from TOJ/BFV.

Second, our examination of the individual fitted data clearly indicated that some participants could not successfully make TOJs for BFD, PLD and FV. R^2^ values (which represent the goodness-of-fit between data and fitted function) below 0.5 were regarded as indicating that participants were unable to achieve a task/stimulus combination (e.g., SJ/BF, TOJ/FV etc.). This criterion was applied to the data of each participant and task/stimulus combination separately and each data set with R^2^ below 0.5 was excluded from the group analysis (for similar exclusion criteria see [8], [20], [24], [25], [27]). Using this criterion, 55 out of 280 data sets (28 participants ×5 stimulus types ×2 tasks) were excluded from the group analysis. Only 1 data set was excluded from SJ conditions, whereas 54 were excluded from TOJ conditions (Table 1). Figure 2 provides examples of excluded individual participant TOJ data for BFD, PLD and FV as well as the means of those participants excluded from these conditions (see also Figures S1 and S2). Examination of the excluded data (Figures 2, S1 and S2) indicates that for many COA levels participants' responses were somewhat random or biased towards one response option, which indicates that those participants were unable to achieve the task. The number of participants excluded from TOJ conditions was not equal across stimulus types: 100% from BFD, 67.9% from PLD, 21.4% from FV, 3.5% from BFV and 0% from BF (Table 1).

**Table 1:** Mean PSS and TIW for both tasks and all stimulus types.

	SJ					TOJ				
	BF	BFV	BFD	PLD	FV	BF	BFV	BFD	PLD	FV
**N**	28	28	28	27	28	28	27	0	9	22
**Excluded (%)**	0	0	0	3.5	0	0	3.5	100	67.9	21.4
**PSS (ms) [s.e.m]**	61 [8.0]	87 [8.7]	38 [6.1]	70 [5.1]	20 [10.8]	−52 [16.6]	−43 [8.8]		−50 [18.7]	−95 [26.1]
**TIW (ms) [s.e.m]**	188 [9.6]	164 [9.2]	128 [7.9]	138 [5.9]	207 [10.4]	146 [13.4]	138 [13.8]		220 [57.7]	279 [30.3]

PSS  =  point of subjective simultaneity. TIW  =  temporal integration window. N  =  number of participants included in group analysis. s.e.m  =  standard error of mean.

Third, to further explore differences in sensitivity to COA between the tasks, repeated measures analysis of variance (ANOVA) tests were conducted on mean TIW data (Table 1) independently for each stimulus type (excluding BFD). As the TIW is derived from the standard deviation of fitted functions it measures how sensitive task responses are to changes in COA, i.e., narrow TIWs represent higher sensitivity to deviation from perceived cue synchrony. The FV TIW was significantly wider for TOJ than SJ (F_1, 21_ = 5.23, p = 0.03), whereas it was significantly narrower for BF (F_1, 27_ = 8.76, p = 0.006). There were no significant differences in TIW between the tasks for either BFV (F_1, 26_ = 2.99, p = 0.09) or PLD (F_1, 8_ = 2.021, p = 0.193). Note that differences in the degrees of freedom across these ANOVAs reflect the fact that only those participants who were able to achieve *both* tasks for a particular stimulus type were included in the ANOVA.

### Mean SJ and TOJ PSS Results

**Figure 3:**
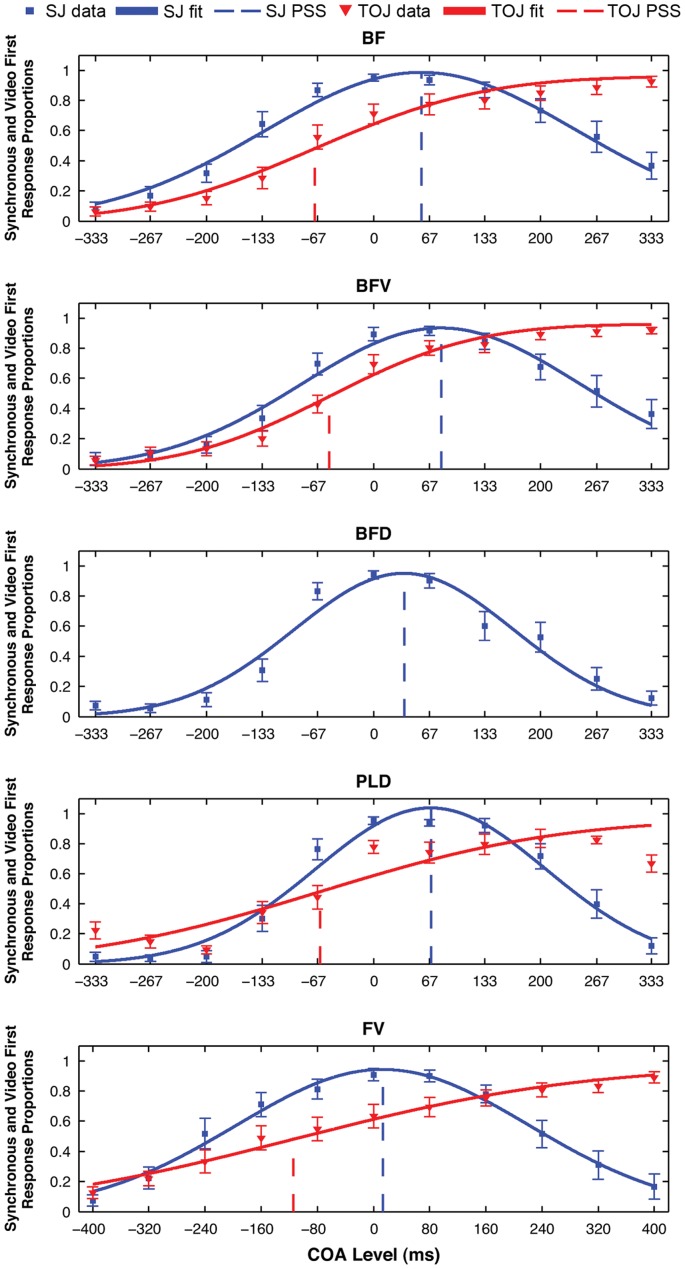
Mean response proportions and fitted functions. Each graph presents data from a different stimulus type, defined in the graph title. Mean proportion of synchronous responses (blue squares for SJ) and video first responses (red triangles for TOJ) are plotted for each COA level along with their corresponding best fitting Gaussian functions. The PSS derived from each fit is indicated by appropriately coloured (blue  =  SJ, red  =  TOJ) vertical dashed lines. Errorbars are ± one standard error of the mean.

Our second hypothesis stated that TOJ PSSs would be audio-leading, while SJ PSSs would be video-leading. In order to test this hypothesis, mean response proportions for each COA level and best-fitting Gaussian curves were calculated using all non-excluded data (Figure 3 and Table 1). Confirming this hypothesis, mean PSSs for all included TOJ/stimulus combinations (red dashed vertical lines in Figure 3) are audio-leading values (negative COA), whereas all SJ/stimulus combinations (blue dashed vertical lines) are video-leading values (positive COA). Two-tailed one-sample t-tests were used to test whether each mean PSS was significantly different from zero (physical synchrony). The PSS of every task/stimulus combination (not including TOJ/BFD) was significantly different from zero (all p<0.027) except for SJ/FV (t_27_ = 1.87, p = 0.072). While the mean data for TOJ/FV and TOJ/PLD are presented in Figure 3 and Table 1, we strongly emphasise, for PLD in particular, that due to the exclusion of participants not able to do these task/stimulus combinations the means are biased estimators of average performance and hence should be interpreted with caution.

### Did PSS or TIW correlate across task and/or stimulus type?

**Figure 4:**
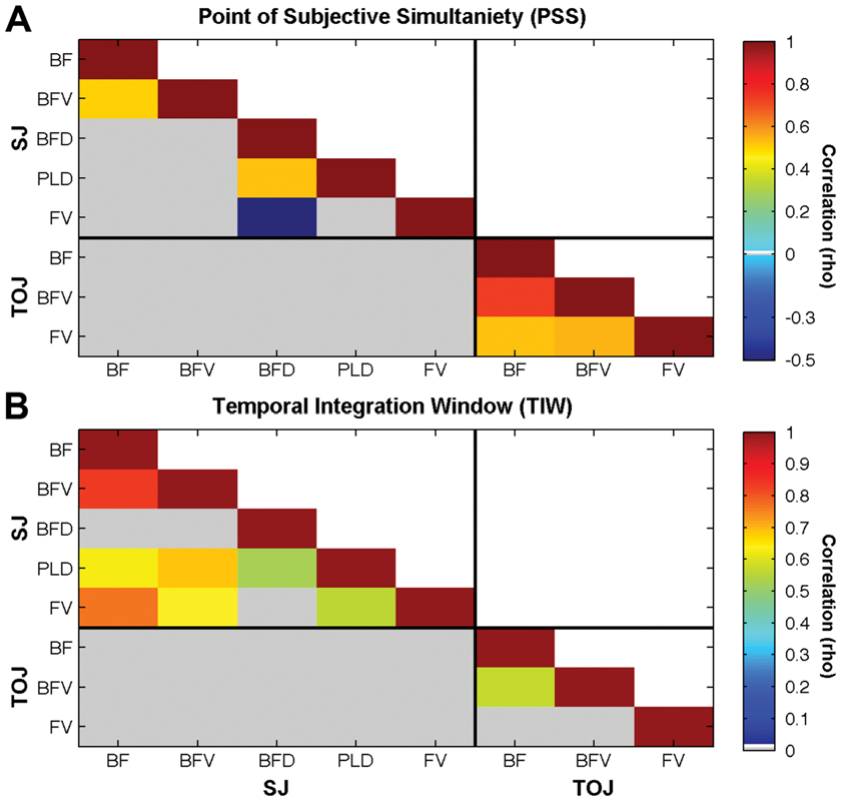
Correlation across stimulus type and task for PSS (**4A**) **and TIWs** (**4B**)**.** Significant Spearman correlations (p<0.05) are displayed as coloured boxes (representing rho correlation values), while non-significant correlations were greyed out.

Our third hypothesis stated that neither PSS nor TIW values would correlate across the two tasks. This hypothesis was confirmed by the striking result that there were no significant Spearman correlations for either PSS or TIW across tasks, i.e., within participants there was no association between the PSS or TIW for any stimulus type in the SJ task and the same parameter for the same stimulus from the TOJ task. In Figures 4A and 4B non-significant Spearman correlations are greyed out, while coloured boxes represent significant (p<0.05) PSS or TIW correlations, respectively. As they had large data exclusion rates, no data from the TOJ/BFD or TOJ/PLD combinations were included in the correlations. Hence, the correlations were calculated from the data of the 22 participants who were able to do SJs for all stimulus types and TOJs for BF, BFV and FV conditions. As well as between task correlations it is also interesting to explore the within task correlations.

All included TOJ PSSs were significantly correlated with each other. For SJ PSSs, the related stimulus conditions of BF and BFV as well as BFD and PLD were positively correlated, while BFD and FV were negatively correlated (Figure 4A). TOJ TIWs for BF and BFV were positively correlated, whereas neither was correlated with FV (Figure 4B). There were positive correlations between TIWs for all SJ/stimulus type combinations except for BFD, which was interestingly only correlated with PLD.

### Audio-leading vs video-leading asynchrony

**Figure 5:**
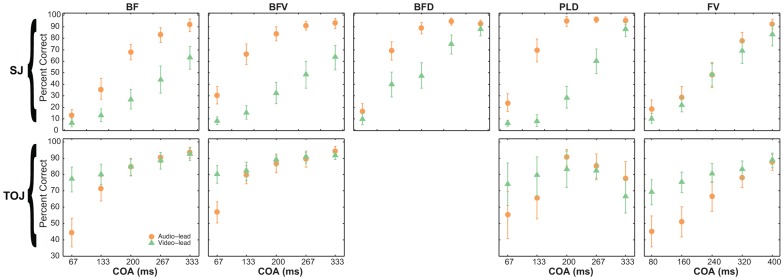
Mean percentage correct for audio-leading and video-leading asynchrony levels. Top and bottom rows display the SJ/stimulus and TOJ/stimulus combinations, respectively. Each graph presents the mean percentage correct for audio leading (orange) and video leading (green) conditions at each cue onset asynchrony (COA). Errorbars represent 95% confidence intervals calculated from 10,000 bootstraps.

It is a classical result in synchrony perception research for participants to be better at detecting audio-leading asynchrony than video-leading (e.g, [15]). A bootstrap analysis showed that for all SJ/stimulus combinations except FV, participants were significantly better at audio-leading asynchrony detection (higher percentage correct in Figure 5). Surprisingly, participants showed no difference in sensitivity between audio-leading and video-leading asynchrony conditions for the SJ/FV combination. Results from the TOJ/stimulus combinations (Figure 5) were very different: overall there were fewer significant differences, and where they did occur participants were better at detecting video-leading than audio-leading cue order. For BF and BFV participants were significantly better at detecting video-leading conditions for the smallest COA level (67 ms), whereas for the other levels there were no significant differences. No significant differences were found at any COA level for the PLD stimuli. For FV video-leading stimuli were detected significantly more often than audio-leading for both the 80 and 160 ms COA levels.

### Point-light-drumming compared to beep-flash-drumming

We also compared the PLD and BFD stimulus conditions to examine what effect having natural human movement information in a point-light-display [40] has on the perception of synchrony. A repeated measures ANOVA, using SJ data, indicated that the mean PSS for BFD (38 ms) was significantly lower (closer to physical synchrony) than the mean PLD PSS (70 ms, F_1, 26_ = 28.07, p<0.001). The same analysis on the TIW data found no difference (F_1, 26_ = 2.51, p = 0.13), which indicates that participants' SJ performance was similar for both drum beat stimulus types but that they may have been expecting longer auditory delay for the more natural point-light-drumming. As no participants were able to do the TOJ/BFD combination it was not possible to conduct the same analysis on TOJ data. It is interesting, however, that the natural human movement in the PLD stimuli enabled some (N = 9) participants to make TOJs for this stimulus.

## Discussion

To highlight differences in the perceptual mechanisms used to achieve SJs and TOJs, the way in which the same group of participants performed on each task was compared for a variety of different stimulus types. To compare the tasks three hypotheses were explored. First, we expected less sensitivity to COA in TOJs than SJs; second, we expected TOJ PSSs to be audio-leading and SJ PSSs to be video-leading and; finally, we expected no correlation between TOJs and SJs on the two most widely used performance measures in synchrony perception research: PSS and TIW. In line with previous work [2], [3], [7]–[10], the psychophysical evidence provided strongly indicates that TOJs and SJs are indeed supported by different perceptual mechanisms for all stimulus types tested.

The first hypothesis of a lower sensitivity to COAs in TOJs than SJs was explored using three measures: a subjective appreciation of task difficulty, data exclusion rates, and the TIW. Interestingly, these three measures provided mixed results. First, for all stimulus types the majority of participants reported the TOJ task to be subjectively the most difficult. Second, exclusion rates were considerably larger for TOJs than SJs for BFD, PLD and FV, but not the other stimulus types. Third, amongst the four stimulus types for which we could compare the TIW across tasks, only for FV did we find larger TIWs for TOJs and, interestingly, participants actually had significantly narrower TIWs for TOJ/BF than for SJ/BF, i.e., they were *more* sensitive to COA for TOJs than SJs.

Using the same stimuli and participants, our results confirm what previous studies have found using different experiments and participants, showing that TIWs for speech are wider during TOJs than SJs [17], [18]. Also comparing TIWs, Maier and colleagues [7] found no main effect of task but did find an interaction between task and stimulus type: the TOJ TIW was wider than that of SJ when an unmodified natural speech stimulus was presented but not for other types of modified speech. Despite the TIW being one of the most widely used performance measures in synchrony perception research, studies directly comparing the two tasks have rarely mentioned TIW differences [2], [8]–[10]. It is not clear whether this is because no differences were found or whether they were not explored. Our data indicates that, at least for some stimulus types, this may be due to there being no significant difference in TIWs across the tasks. However, even if comparing TIWs fails to find differences in sensitivity, such differences may still exist, and other outcome measures may capture them. Here, we demonstrated that most participants found the TOJ task to be subjectively more difficult than the SJ task for all the stimulus types presented, plus considerably more participants were unable to successfully achieve TOJs for two of our stimulus types (BFD and PLD), which they could achieve SJs for.

One possible reason why many more TOJ data sets had to be excluded for BFD and PLD than for BF, BFV and to a lesser extent FV is related to the difference in how asynchrony was created for these stimulus types. Due to the overall longer duration of the original PLD stimulus [8], [32], [33], asynchronous conditions could be created for both PLD and BFD by cutting the stimuli from a larger movie sequence, after separating the audio and visual timelines to produce cue asynchrony (middle panel of Figure 1B). This ensured that there was audio and visual information at both the beginning and end of the stimulus and that duration remained a constant 3 seconds for all COA levels [8], [32], [33]. In contrast, BF, BFV, and FV had more finite audio and video sequences, i.e., shorter sequences could not be cut from larger sequences, and separating them to produce asynchrony created gaps between the audio and visual cues at both the beginning and end of the audiovisual stimulus; consequently, stimulus duration increased with increasing COA level (left and right panels of Figure 1B). The information provided by the creation of such gaps may be more salient to making TOJs than SJs; therefore, the lack of such gaps in BFD and PLD may have made it more difficult for participants to successfully make TOJs about those stimuli. In line with this speculation, Maier and colleagues [6] recently found that for speech stimuli participants mainly used the information present at the beginning and end of a sentence to make TOJs, while the full stimulus appeared to be used to make SJs. However, the current data also highlight that factors other than timing characteristics play an important role in TOJs: PLD and BFD had the same onset, offset and duration yet no participants could successfully make TOJs for BFD, while 32% of participants could for PLD. Note, however, that the current experiment focused on using a variety of stimulus types to investigate general differences between SJs and TOJs. In doing so we have also shown that differences between the two tasks are not based on the potential confound that when stimulus duration increases with increasing COA SJs but not TOJs can be achieved by focusing on these duration differences. Our results highlight clear differences between SJs and TOJs for all stimulus types tested including BFD and PLD to which the confound does not apply since their stimulus duration was a constant 3 seconds at all COA levels. Therefore, the current data further indicates that the tasks are supported by different perceptual mechanisms and circumvents a potential confound to similar conclusions previously made [2], [3], [9], [10]. Future studies are required to fully understand the intricacies of how these tasks differ, for example, in their use of specific stimulus properties and their potentially different supporting neural mechanisms.

We propose that, in the context of a within subjects design, differences in exclusion rates between SJs and TOJs about the same stimulus type can be used as an outcome measure. Although we are not aware of this having been done before in synchrony perception research, it is common for data to be flagged as noisy and excluded (e.g., [17], [19]–[28], [29]–[31]). Moreover, such exclusion rates vary widely, even under very similar experimental settings and using the same stimulus type [22], [24], [26]. Our proposal relies strongly on interpreting data exclusion as reflecting a participant's inability to achieve the task for the COA levels presented. However, it could be argued that it simply reflects the result of fitting an inappropriate psychometric function to the data. This alternative interpretation cannot conclusively be ruled out, however, observation of the excluded TOJ data showed that the exclusion resulted from the participants not achieving the task properly, either because their responses were random or because they were biased toward one response (Figures 2, S1 and S2). Such responses indicate a lack of sensitivity to COA rather than an inappropriate fitting. Attempting to fit them with the appropriate psychometric function for the task produces a relatively flat function with a large standard deviation and ultimately an unacceptable goodness-of-fit. Petrini and colleagues (Figure 4b of [8]) presented the only other illustrations of excluded data that we are aware of, and also found similar patterns of random and biased TOJ responses to a PLD stimulus from some participants. If we assume therefore, that not being able to fit a participant's data represents a lack of sensitivity to the independent variable and hence an inability to achieve the task, then data removal can be regarded as an outcome measure rather than simply noisy data that should be removed from further analysis.

Overall, the evidence presented does not allow us to definitively confirm our hypothesis that participants are less sensitive to COA during a TOJ task than during an SJ task. In fact, significantly smaller TIWs for TOJ/BF than SJ/BF indicate the opposite effect. However, we have highlighted that there are subjective task demand differences between TOJs and SJs for all stimulus types presented, and, for some stimulus types at least, participants are less sensitive to COA when trying to discriminate cue order as opposed to when attempting to discriminate synchrony from asynchrony. We propose that mean TIWs calculated after excluding noisy data, while informative, are not sufficient to successfully capture differences in task demands between TOJs and SJs and that other measures are required to do so.

Despite insufficient evidence to support hypothesis 1, significant support for both hypotheses 2 and 3 provides strong, converging evidence [2], [3], [7]–[10] that TOJs and SJs involve different perceptual mechanisms. Confirming our second hypothesis, that TOJ PSSs would be audio-leading while SJ PSSs would be video-leading, the PSS of every TOJ/stimulus combination was indeed an audio-leading COA, while the PSS of every SJ/stimulus combination was a video-leading COA. This result reflects the overall summary of previous synchrony perception research provided by Van Eijk et al. [2], that average audio-leading PSSs are “almost exclusively” reported for TOJs, whereas SJ average PSSs are generally video-leading.

In support of our third hypothesis, that neither PSS nor TIW values would correlate across the two tasks, no significant association was found across the tasks for either of these measures. A lack of correlation between TOJs and SJs had previously been reported for other audiovisual stimuli [2], [9] and for a variety of other crossmodal stimuli [3]. If SJs and TOJs resulted from the same perceptual mechanism we would expect an association between the performance measures derived from them. The evidence presented here clearly shows that there is no such association. Moreover, training on one of the tasks would be expected to transfer to the other if they shared the same perceptual mechanism; however, this transfer of training does not occur [41]. Therefore, this converging evidence across different hypotheses and experiments supports the conclusion that the perceptual mechanisms of SJs and TOJs are different.

One surprising result of the current experiment concerned the SJ/FV combination, in which participants were not better at detecting audio-leading asynchrony than video-leading. It is a classical result in psychophysics literature that in SJ tasks, participants are much better at detecting asynchrony when the audio cue leads the visual compared to the other way round [2], [8], [29], [32], [33], [35], [42]. We again provide evidence for this classical effect in all stimulus types except FV (Figure 5). Not finding this effect for the FV stimuli is surprising and inconsistent with previous work using speech stimuli [15], [30], [31], [43]. Furthermore, in a separate unpublished study with the same speech stimuli as used here, we did find that participants were better at audio-leading detection during an SJ task. The only differences between that study and the current experiment are different participants, and that here participants completed SJ and TOJ blocks in the same experimental run. Note that none of the other related work, just discussed, had this task switching in the same experimental run either. Therefore, the task switching in the current experiment may have influenced participants' SJs for FV stimuli, the result of which may have been no difference in sensitivity to asynchrony between audio- and video-leading conditions. If this is true, however, the same task switching did not influence SJ or TOJ performance for PLD, as the results of this stimulus type were consistent with previous data using the same stimuli without a task switching design [8], [32], [33]. Looking for evidence of this commonly found effect (better audio-leading detection than video-leading) across the different tasks provided more evidence that SJs and TOJs should not be regarded as representing the same underlying process of synchrony perception. Participants were not better at audio-leading detection during the TOJ task for any stimulus type; in fact, when they differed at all (mainly at lower COA levels), audio-leading performance was actually worse than video-leading (Figure 5).

Neuroscientists have used manipulations of temporal synchrony to explore synchrony perception and multisensory integration in general [1]. For example, several functional magnetic resonance imaging (fMRI) studies have explored how the brain responds to synchronous and asynchronous versions of audiovisual stimuli (e.g., [34], [44]–[47]). There are implications from the results of the current study for the interpretation of such experiments and for the design of future neuroimaging work. It is not uncommon for participants in these fMRI experiments to be asked to perform either an orthogonal task, i.e., one not related to synchrony, or even no task at all [34], [45]. This could be problematic, as under such experimental settings there is no evidence as to whether participants focused on the temporal order or the simultaneity/successiveness of the cues; it is even possible that different participants may have focused on different factors or that they changed their focus throughout. Since our results clearly indicate that SJs and TOJs are supported by different perceptual mechanisms, it is important to control the task performed by participants to ensure their attention is focused on the same factor for the entire experiment. The best way to do this is to give participants a specific task related to synchrony perception, potentially a TOJ, an SJ or alternative tasks previously outlined (e.g., [8]). What is most important is that whichever task is chosen, in either neuroimaging or psychophysics studies, the results should be interpreted in relation to the task and stimulus; furthermore, synchrony perception has many factors and a single task is most likely not sufficient to explore them [2]. Finally, an interesting question raised by the current psychophysics study is whether neuroimaging techniques can be used to define the location and functional properties of the different mechanisms supporting these two tasks.

## Supporting Information

Figure S1 **Randomly selected examples of excluded BFD data.**
(TIF)

Figure S2 **Randomly selected examples of excluded PLD data.**
(TIF)

Movie S1 **Example BF stimulus.**
(MOV)

Movie S2 **Example BFV stimulus.**
(MOV)

Movie S3 **Example BFD stimulus.**
(MOV)

Movie S4 **Example PLD stimulus.**
(MOV)

Movie S5 **Example FV stimulus.**
(MOV)

## References

[1] Stein BE, Meredith MA (1993) The Merging of the Senses. Cambridge, MA: MIT Press. 224 p.

[2] Van Eijk RLJ, Kohlrausch A, Juola JF, Van De Par S (2008) Audiovisual synchrony and temporal order judgments: effects of experimental method and stimulus type. Perception & psychophysics 70: 955–68 doi:10.3758/PP.70.6.955. 18717383 10.3758/pp.70.6.955

[3] Fujisaki W, Nishida S (2009) Audio-tactile superiority over visuo-tactile and audio-visual combinations in the temporal resolution of synchrony perception. Experimental brain research 198: 245–59 doi:10.1007/s00221-009-1870-x. 19499212 10.1007/s00221-009-1870-x

[4] Fujisaki W, Nishida S (2007) Feature-based processing of audio-visual synchrony perception revealed by random pulse trains. Vision research 47: 1075–93 doi:10.1016/j.visres.2007.01.021. 17350068 10.1016/j.visres.2007.01.021

[5] Hirsh IJ (1959) Auditory Perception of Temporal Order. The Journal of the Acoustical Society of America 31: 759 doi:10.1121/1.1907782.

[6] Allan LG (1975) The relationship between judgments of successiveness and judgments of order. Perception & Psychophysics 18: 29–36 doi:10.3758/BF03199363.

[7] Maier JX, Luca M Di, Noppeney U (2011) Audiovisual asynchrony detection in human speech. Journal of experimental psychology. Human perception and performance 37: 245–256 doi:10.1037/a0019952. 20731507 10.1037/a0019952

[8] Petrini K, Holt SP, Pollick FE (2010) Expertise with multisensory events eliminates the effect of biological motion rotation on audiovisual synchrony perception. Journal of Vision 10(5): 1–14 doi:10.1167/10.5.2. 10.1167/10.5.2 20616132

[9] Vatakis A, Navarra J, Soto-Faraco S, Spence C (2008) Audiovisual temporal adaptation of speech: temporal order versus simultaneity judgments. Experimental brain research 185: 521–9 doi:10.1007/s00221-007-1168-9. 17962929 10.1007/s00221-007-1168-9

[10] Vroomen J, Stekelenburg JJ (2011) Perception of intersensory synchrony in audiovisual speech: not that special. Cognition 118: 75–83 doi:10.1016/j.cognition.2010.10.002. 21035795 10.1016/j.cognition.2010.10.002

[11] Chandrasekaran C, Trubanova A, Stillittano S, Caplier A, Ghazanfar AA (2009) The natural statistics of audiovisual speech. PLoS computational biology 5: e1000436 doi:10.1371/journal.pcbi.1000436. 19609344 10.1371/journal.pcbi.1000436 PMC2700967

[12] Sams M, Aulanko R, Hämäläinen M, Hari R, Lounasmaa OV, et al (1991) Seeing speech: visual information from lip movements modifies activity in the human auditory cortex. Neuroscience letters 127: 141–5. 1881611 10.1016/0304-3940(91)90914-f

[13] Schroeder CE, Lakatos P, Kajikawa Y, Partan S, Puce A (2008) Neuronal oscillations and visual amplification of speech. Trends in cognitive sciences 12: 106–13 doi:10.1016/j.tics.2008.01.002. 18280772 10.1016/j.tics.2008.01.002 PMC3987824

[14] Van Wassenhove V, Grant KW, Poeppel D (2005) Visual speech speeds up the neural processing of auditory speech. Proceedings of the National Academy of Sciences of the United States of America 102: 1181–6 doi:10.1073/pnas.0408949102. 15647358 10.1073/pnas.0408949102 PMC545853

[15] Dixon NF, Spitz L (1980) The detection of auditory visual desynchrony. Perception 9: 719–21. 7220244 10.1068/p090719

[16] Vatakis A, Spence C (2006) Audiovisual synchrony perception for music, speech, and object actions. Brain research 1111: 134–42 doi:10.1016/j.brainres.2006.05.078. 16876772 10.1016/j.brainres.2006.05.078

[17] Soto-Faraco S, Alsius A (2009) Deconstructing the McGurk-MacDonald illusion. Journal of experimental psychology. Human perception and performance 35: 580–7 doi:10.1037/a0013483. 19331510 10.1037/a0013483

[18] Soto-Faraco S, Alsius A (2007) Conscious access to the unisensory components of a cross-modal illusion. Neuroreport 18: 347–50 doi:10.1097/WNR.0b013e32801776f9. 17435600 10.1097/WNR.0b013e32801776f9

[19] Bertelson P, Aschersleben G (2003) Temporal ventriloquism: crossmodal interaction on the time dimension 1. Evidence from auditory-visual temporal order judgment. International Journal of Psychophysiology 50: 147–155 doi:10.1016/S0167-8760(03)00130-2. 14511842 10.1016/s0167-8760(03)00130-2

[20] Boenke LT, Deliano M, Ohl FW (2009) Stimulus duration influences perceived simultaneity in audiovisual temporal-order judgment. Experimental brain research 198: 233–44 doi:10.1007/s00221-009-1917-z. 19590862 10.1007/s00221-009-1917-z

[21] Koppen C, Spence C (2007) Audiovisual asynchrony modulates the Colavita visual dominance effect. Brain research 1186: 224–32 doi:10.1016/j.brainres.2007.09.076. 18005944 10.1016/j.brainres.2007.09.076

[22] Santangelo V, Spence C (2009) Crossmodal exogenous orienting improves the accuracy of temporal order judgments. Experimental brain research 194: 577–86 doi:10.1007/s00221-009-1734-4. 19242685 10.1007/s00221-009-1734-4

[23] Van der Burg E, Olivers CNL, Bronkhorst AW, Theeuwes J (2008) Audiovisual events capture attention: evidence from temporal order judgments. Journal of vision 8: 1–10 doi:10.1167/8.5.2. 10.1167/8.5.2 18842073

[24] Zampini M, Shore DI, Spence C (2003) Multisensory temporal order judgments: the role of hemispheric redundancy. International Journal of Psychophysiology 50: 165–180 doi:10.1016/S0167-8760(03)00132-6. 14511844 10.1016/s0167-8760(03)00132-6

[25] Zampini M, Shore DI, Spence C (2003) Audiovisual temporal order judgments. Experimental brain research 152: 198–210 doi:10.1007/s00221-003-1536-z. 12879178 10.1007/s00221-003-1536-z

[26] Vatakis A, Bayliss L, Zampini M, Spence C (2007) The influence of synchronous audiovisual distractors on audiovisual temporal order judgments. Perception & psychophysics 69: 298–309. 17557599 10.3758/bf03193751

[27] Spence C, Shore DI, Klein RM (2001) Multisensory prior entry. Journal of Experimental Psychology: General 130: 799–832 doi:10.1037/0096-3445.130.4.799. 11757881 10.1037//0096-3445.130.4.799

[28] Alais D, Cass J (2010) Multisensory Perceptual Learning of Temporal Order: Audiovisual Learning Transfers to Vision but Not Audition. PLoS ONE 5: e11283 doi:10.1371/journal.pone.0011283. 20585664 10.1371/journal.pone.0011283 PMC2890588

[29] Stone JV, Hunkin NM, Porrill J, Wood R, Keeler V, et al (2001) When is now? Perception of simultaneity. Proceedings. Biological sciences/The Royal Society 268: 31–38 doi:10.1098/rspb.2000.1326. 10.1098/rspb.2000.1326 PMC1087597 12123295

[30] Van Wassenhove V, Grant KW, Poeppel D (2007) Temporal window of integration in auditory-visual speech perception. Neuropsychologia 45: 598–607 doi:10.1016/j.neuropsychologia.2006.01.001. 16530232 10.1016/j.neuropsychologia.2006.01.001

[31] Conrey B, Pisoni DB (2006) Auditory-visual speech perception and synchrony detection for speech and nonspeech signals. The Journal of the Acoustical Society of America 119: 4065 doi:10.1121/1.2195091. 16838548 10.1121/1.2195091 PMC3314884

[32] Petrini K, Dahl S, Rocchesso D, Waadeland CH, Avanzini F, et al (2009) Multisensory integration of drumming actions: musical expertise affects perceived audiovisual asynchrony. Experimental brain research 198: 339–52 doi:10.1007/s00221-009-1817-2. 19404620 10.1007/s00221-009-1817-2

[33] Petrini K, Russell M, Pollick FE (2009) When knowing can replace seeing in audiovisual integration of actions. Cognition 110: 432–9 doi:10.1016/j.cognition.2008.11.015. 19121519 10.1016/j.cognition.2008.11.015

[34] Stevenson RA, Altieri NA, Kim S, Pisoni DB, James TW (2010) Neural processing of asynchronous audiovisual speech perception. NeuroImage 49: 3308–18 doi:10.1016/j.neuroimage.2009.12.001. 20004723 10.1016/j.neuroimage.2009.12.001 PMC2818746

[35] Arrighi R, Alais D, Burr D (2006) Perceptual synchrony of audiovisual streams for natural and artificial motion sequences. Journal of vision 6: 260–8 doi:10.1167/6.3.6. 16643094 10.1167/6.3.6

[36] Brainard DH (1989) Calibration of a computer controlled color monitor. Color Research & Application 14: 23–34 doi:10.1002/col.5080140107.

[37] Pelli D (1997) The VideoToolbox software for visual psychophysics: transforming numbers into movies. Spatial Vision 10: 437–442. 9176953

[38] Vatakis A, Spence C (2006) Audiovisual synchrony perception for speech and music assessed using a temporal order judgment task. Neuroscience letters 393: 40–4 doi:10.1016/j.neulet.2005.09.032. 16213656 10.1016/j.neulet.2005.09.032

[39] Wilcox RR (2005) Introduction to Robust Estimation and Hypothesis Testing. 2nd Editio. San Diago, CA: Academic Press. 608 p.

[40] Johansson G (1973) Visual perception of biological motion and a model for its analysis. Perception & Psychophysics 14: 201–211.

[41] Mossbridge JA, Fitzgerald MB, O'Connor ES, Wright BA (2006) Perceptual-learning evidence for separate processing of asynchrony and order tasks. The Journal of neuroscience 26: 12708–16 doi:10.1523/JNEUROSCI.2254-06.2006. 17151274 10.1523/JNEUROSCI.2254-06.2006 PMC6674828

[42] Lewald J, Guski R (2003) Cross-modal perceptual integration of spatially and temporally disparate auditory and visual stimuli. Cognitive brain research 16: 468–78. 12706226 10.1016/s0926-6410(03)00074-0

[43] Grant KW, Van Wassenhove V, Poeppel D (2004) Detection of auditory (cross-spectral) and auditory-visual (cross-modal) synchrony. Speech Communication 44: 43–53 doi:10.1016/j.specom.2004.06.004.

[44] Miller LM, D'Esposito M (2005) Perceptual fusion and stimulus coincidence in the cross-modal integration of speech. The Journal of neuroscience 25: 5884–93 doi:10.1523/JNEUROSCI.0896-05.2005. 15976077 10.1523/JNEUROSCI.0896-05.2005 PMC6724802

[45] Noesselt T, Rieger JW, Schoenfeld MA, Kanowski M, Hinrichs H, et al (2007) Audiovisual temporal correspondence modulates human multisensory superior temporal sulcus plus primary sensory cortices. The Journal of neuroscience 27: 11431–41 doi:10.1523/JNEUROSCI.2252-07.2007. 17942738 10.1523/JNEUROSCI.2252-07.2007 PMC2957075

[46] Lewis R, Noppeney U (2010) Audiovisual synchrony improves motion discrimination via enhanced connectivity between early visual and auditory areas. The Journal of neuroscience 30: 12329–39 doi:10.1523/JNEUROSCI.5745-09.2010. 20844129 10.1523/JNEUROSCI.5745-09.2010 PMC6633449

[47] Petrini K, Pollick FE, Dahl S, McAleer P, McKay L, et al (2011) Action expertise reduces brain activity for audiovisual matching actions: An fmri study with expert drummers. NeuroImage 56: 1480–1492 doi:10.1016/j.neuroimage.2011.03.009. 21397699 10.1016/j.neuroimage.2011.03.009

