# Supplementary figures and images for: A Psychophysical Investigation of Differences between Synchrony and Temporal Order Judgments
Source: PLoS One. 2013 Jan 21;8(1):e54798. doi: 10.1371/journal.pone.0054798 (PMC3549984; doi:10.1371/journal.pone.0054798)

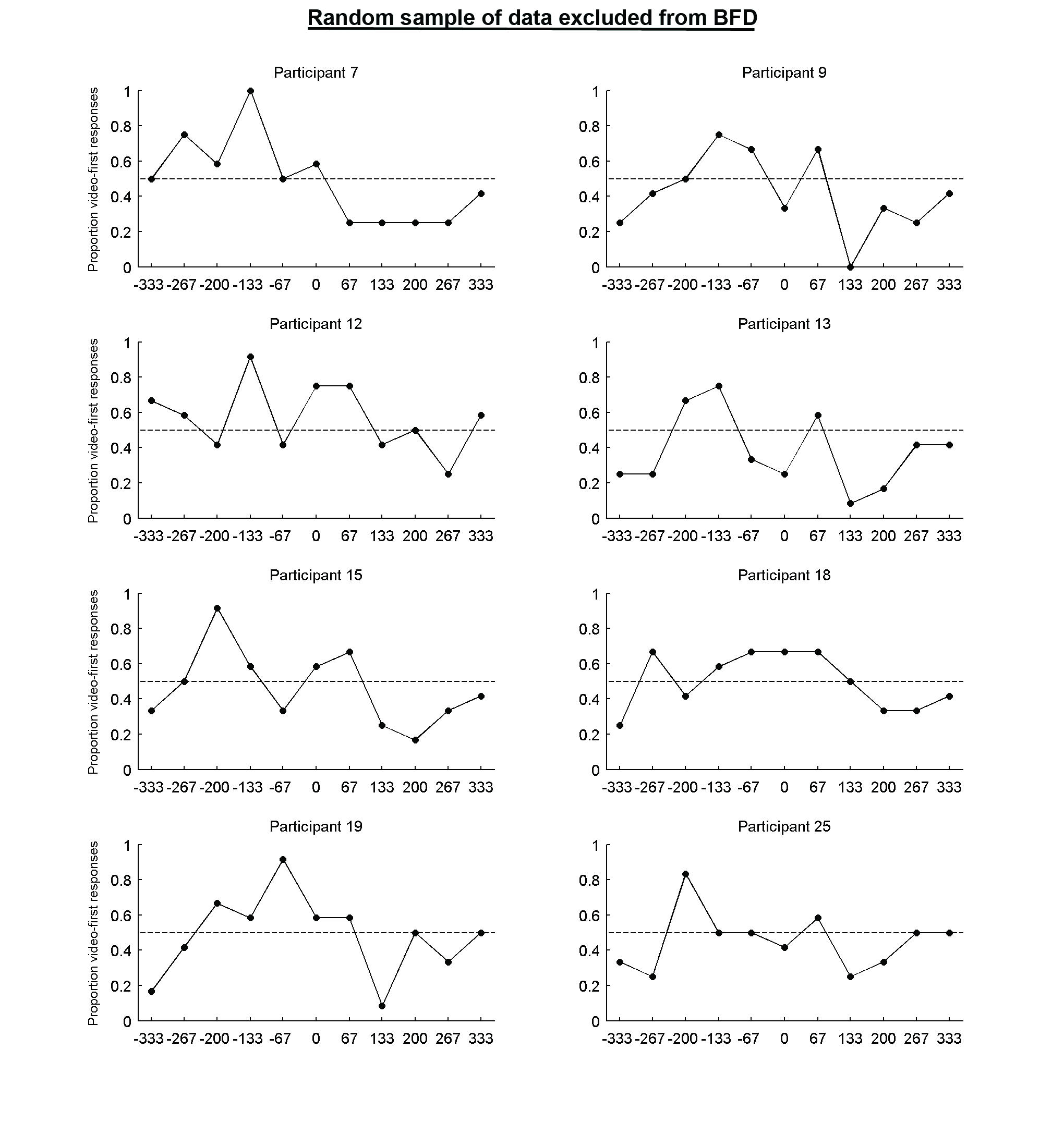

Supplement: Figure S1 — Randomly selected examples of excluded BFD data. (TIF) [file pone.0054798.s001.tif]

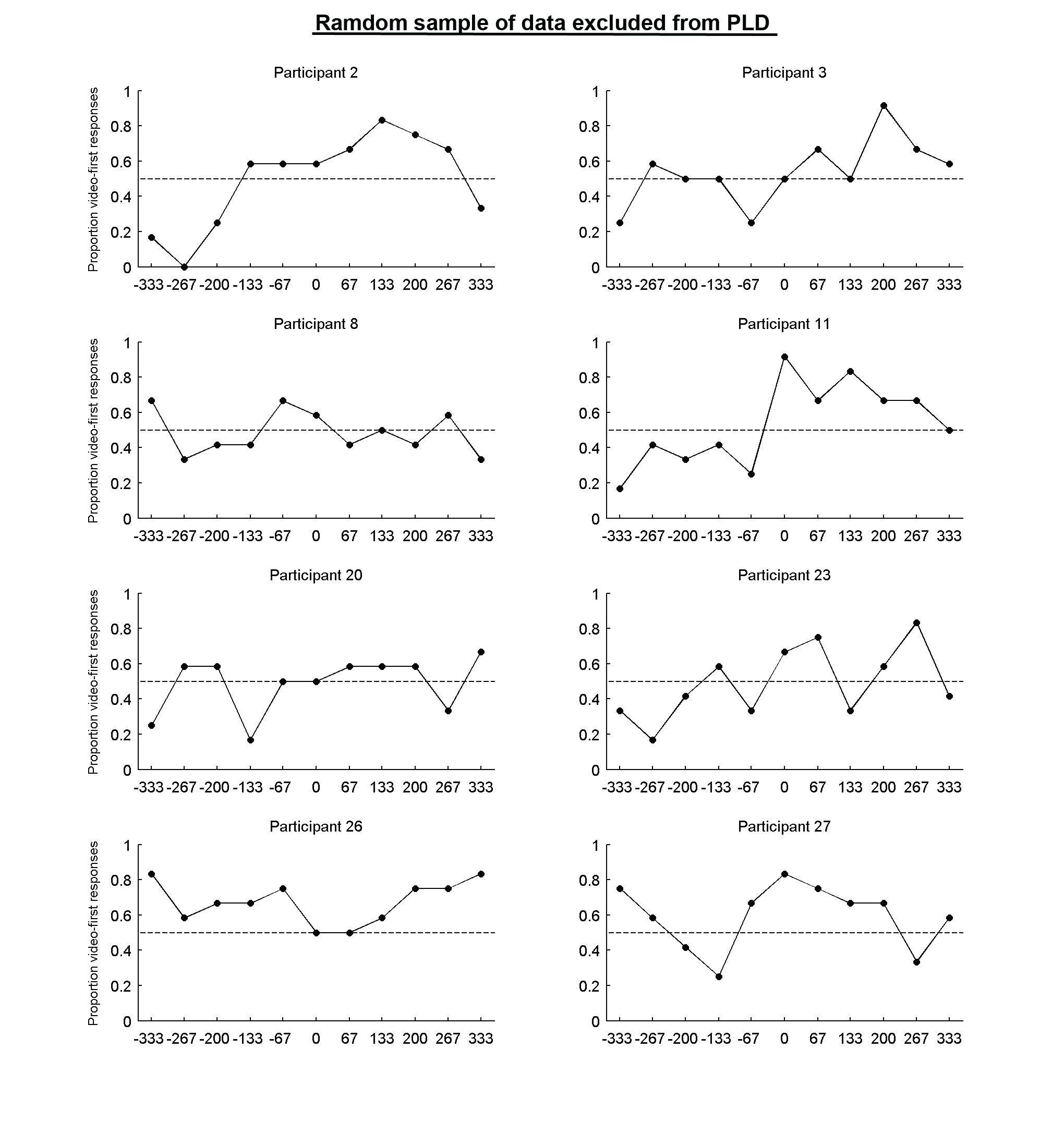

Supplement: Figure S2 — Randomly selected examples of excluded PLD data. (TIF) [file pone.0054798.s002.tif]
